# Multiple Epithelioid Hemangiomas with Orbital Involvement

**DOI:** 10.1155/2015/629805

**Published:** 2015-01-31

**Authors:** Branislava Miličić, Tomaž Velnar, Rado Pregelj, Clara Limbaeck-Stokin

**Affiliations:** ^1^Faculty of Medicine, Institute of Pathology, University of Ljubljana, 1000 Ljubljana, Slovenia; ^2^Department of Neurosurgery, University Medical Center Maribor, 2000 Maribor, Slovenia; ^3^International Institute for Neurosurgery and Neurosciences, 1000 Ljubljana, Slovenia

## Abstract

Epithelioid hemangioma, also known as angiolymphoid hyperplasia with eosinophilia, is a cutaneous angioproliferative lesion that follows a benign clinical course. It is most frequently localized in the skin of the head and neck region; although it may sometimes arise deeper in soft tissues, orbital involvement is rare. Here we describe a patient who developed multiple epithelioid hemangiomas, including an intraorbital lesion. The histopathological parallels with other reactive and neoplastic lesions as well as therapeutic options are discussed.

## 1. Introduction

Epithelioid hemangioma (EH) is an uncommon vasoproliferative process of the skin and less frequently soft tissues that usually affects middle-aged adults [1]. EH typically presents as painless, erythematous nodules or plaques of the head and neck region, involving the dermis and subcutaneous tissue. The process tends to recur and roughly 50% of affected patients develop multiple lesions [1, 2]. Despite these worrisome features the clinical course of EH is benign; pathogenesis though remains unclear and its nature, whether it is a reactive process or a neoplasm, is still a matter of discussion.

A definitive diagnosis requires histological evaluation: EH has distinctive morphological features consisting of proliferation of blood vessels lined by abnormal endothelial cells, accompanied by lymphocytic and eosinophilic inflammatory infiltrate [2, 3]. Importantly, the endothelial cells are plump but do not show signs of atypia or increased mitotic activity [4]. Orbital involvement is very rare and appears to have a later onset. So far only few cases have been reported in the literature [5, 6].

## 2. Case Report

**Figure 1:**
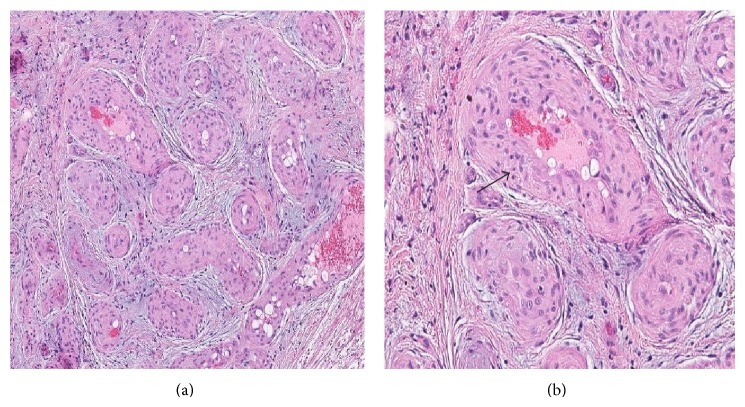
Representative histological images of the first biopsy: (a) HE: low magnification, showing numerous blood vessels. (b) HE: higher magnification, showing thick vessel walls with proliferated endothelium (arrow) and some inflammatory infiltrate.

A 71-year-old woman with a history of sarcoidosis and non-Hodgkin lymphoma of the gastrointestinal tract was admitted to the neurosurgery department for crushing pain in the right eye, headaches, and double vision. A year before, a tumour of the right orbit was partially excised. Histopathological examination at that time showed fibrous tissue with vascular proliferation (Figure 1). The nature of the proliferation, reactive or neoplastic, was not definitively established at that time. Nevertheless the lesion was considered benign because histological parameters typical for malignant neoplasm—high mitotic activity, cellular atypia, necrosis, and infiltrative growth pattern—were not observed; therefore further treatment was not recommended. Symptoms, however, recurred after a few months and were progressively worsening.

At the time of admission to the hospital, exophthalmos of the right eye was evident with paresis of the third and fourth cranial nerves. Visual acuity was 0.8 and had not changed since previous hospitalization. The patient complained of double vision, which was exaggerated in the gaze directions of the affected orbital muscles. No other cranial nerve abnormalities were noted.

**Figure 2:**
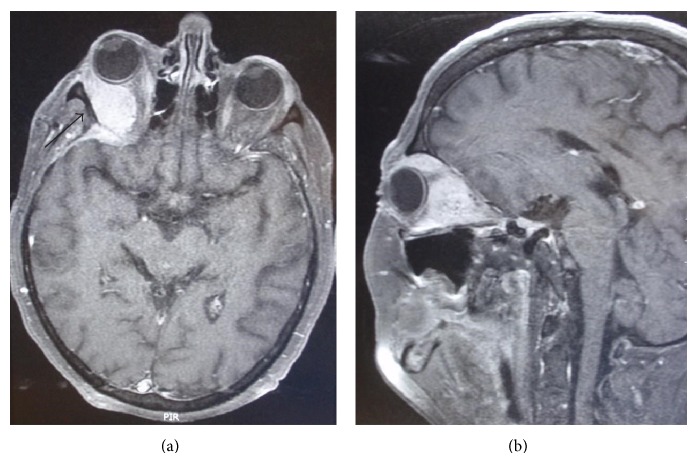
MRI: (a) T2-weighted MRI image of the head showing intraorbital mass and scar tissue (arrow) from previous operation. (b) On contrast T1-weighted image, the intraorbital mass may be clearly seen, as well as exophthalmos.

**Figure 3:**
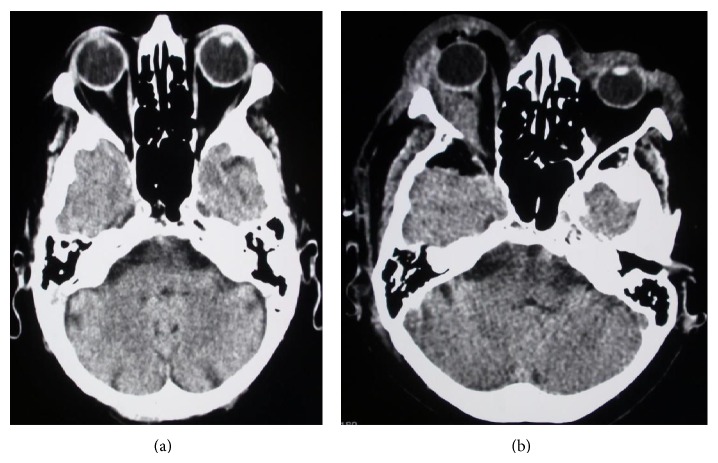
CT, pre- and postoperative: (a) Preoperative CT: intraorbital mass (thick arrow) and partially removed bone of the orbital roof (thin arrow) are seen. (b) Postoperative CT: the intraorbital mass has been removed and the exophthalmos is less clearly evident in comparison to preoperative images.

MRI of the head revealed a mass of the right orbit, about two centimetres in diameter (Figure 2). Some contrast staining was present in the operative field, indicating scar tissue from previous surgery. A CT scan showed a defect of the orbital roof bone, removed at the time of the first operation (Figure 3(a)). Surgical resection was indicated.

Surgery was performed through right pterional approach with retrepanation of the cranial bone and complete orbitotomy. A fibrous tumour was found in the periorbital adipose tissue, embedded in scar tissue and extending to the fibrous coverings of the globe, partially adherent to the extraocular muscles. The tumour was completely resected.

**Figure 4:**
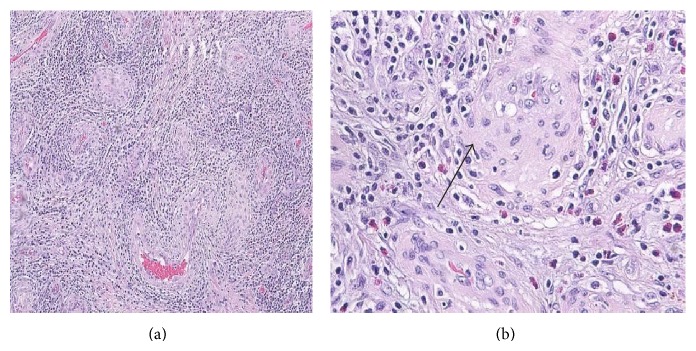
Histological images of the second biopsy: (a) HE: lower magnification, compared to the first biopsy blood vessels are surrounded by a denser inflammatory infiltrate. (b) HE: higher magnification, showing marked endothelial proliferation. Endothelial cells are plump (arrow). Many eosinophils are seen.

After surgery, exophthalmos and ocular pain slowly subsided, as did paresis of the third and fourth cranial nerves, although some minor deficit of ocular motion persisted (Figure 3(b)). Histopathological evaluation revealed a lesion composed of numerous blood vessels and fibrous tissue, accompanied by a dense, mixed inflammatory infiltrate including eosinophils, lymphocytes, and plasma cells. Focal lymphocyte aggregates were also present, while granulomas were not observed. The vascular walls were thick, lined by mildly polymorphic, plump endothelial cells with abundant eosinophilic cytoplasm and vesicular nuclei. Necrosis and mitoses were not seen (Figure 4). In comparison with the first biopsy (Figure 1), there was more inflammatory infiltrate, probably secondary to surgery, blood vessels displayed thicker walls and the epithelioid appearance of the endothelium was more pronounced. Based on these histopathological findings, a diagnosis of EH was made.

In addition, we retrieved two older biopsies of the same patient from our archives. Both were performed two years earlier; the first one was a skin biopsy of the scalp and the second was a biopsy of the right orbital adipose tissue and lacrimal gland. The lesion of the scalp also displayed histopathological features of EH, while interestingly in the lacrimal gland granulomatous inflammation turned out to be consistent with sarcoidosis.

Also, a new tumour localized on the eyelid has developed a few months after surgery. This lesion has not been histologically verified yet, but according to its clinical features, it could represent another EH.

## 3. Discussion

EH was first described in 1969 as angiolymphoid hyperplasia with eosinophilia [7, 8] and, although uncommon, it is now a well-recognised entity both at superficial and deep soft tissue sites, as well as in bones. Orbital involvement is rare, but when present symptoms such as exophthalmos, ocular pain, tearing, pruritus around the eye, blurred vision, and double vision have been described [5, 6]. These last two symptoms were observed in our patient and clearly reflect involvement of ocular cranial nerves.

The orbital tumour exhibited convincing histopathological elements of EH, evident in particular in the second biopsy and closely resembling the lesion previously excised from the scalp. Nevertheless, a differential diagnosis in particular with diseases that more commonly affect the orbit had to be considered. This included reactive processes, lymphoproliferative disorders, and some vascular neoplasms.

Several reactive processes had to be ruled out. In this category, the main differential diagnosis was with Kimura's disease (KD), since EH shares with KD some clinical and histologic features. Both KD and EH usually manifest as nodules or plaques in the head and neck region. KD probably represents an allergic or autoimmune response and, unlike EH, it often develops systemic features such as blood eosinophilia and nephrotic syndrome, asthma, tuberculosis, or Loeffler syndrome [9–11]. KD differs from EH also histologically, as in KD proliferate blood vessels do not display abnormal epithelioid endothelial cells [4].

Based on tumour location, the second major reactive process to be excluded was idiopathic orbital inflammation (IOI). IOI is characterized histologically by a mixed inflammatory infiltrate and in later stages fibrosis, but again abnormal endothelial proliferation is not seen [12].

Since the cellular infiltrate was rich in lymphoid cells, the second group of diseases to enter into the differential diagnosis was lymphoproliferative disorders. This diagnosis was excluded considering the reactive appearance of the infiltrate, which was composed of a mixed population of well-differentiated lymphatic cells.

Vascular tumours represent the third family of lesions that had to be considered. Among benign vascular tumours, especially arteriovenous hemangioma (cirsoid aneurism) had to be taken into account. Cirsoid aneurisms also present as a nodule or papule in the head and neck region of middle-aged or elderly adults. Histologically, they are characterised by a combination of thin and thick-walled blood vessels with no epithelioid endothelium. Malignant vascular neoplasms, in particular epithelioid hemangioendothelioma and angiosarcoma, also had to be excluded [1]; according to the benign histological features of the blood vessels observed in the biopsy (well-formed vessels, no cellular atypia of endothelial cells), this diagnosis was easily ruled out in our case.

The aetiology of EH remains unknown. It is either a benign neoplastic proliferation of blood vessels or a reactive hyperplasia of vascular structures, which develops in response to trauma, and possibly other triggers such as hyperestrogenic states (pregnancy or oral contraceptives) or infection [13–15]. It is interesting in our case that the patient had sarcoidosis with histologically confirmed involvement of the right lacrimal gland. We have not found any association between sarcoidosis and EH in the literature, but we cannot exclude a connection among the two processes considering that EH developed after granulomatous dacryoadenitis.

There are many treatment options for EH depending on the extent, location, and size of the lesions [6]. The most effective therapy is complete removal and follow-up [16]. Possible nonsurgical possible treatments include intralesional injections of isotretinoin, steroids, interferon alpha-2a, cytotoxic agents and irradiation therapy, carbon dioxide and argon laser, and electrodessication [13, 15–17]. Orbital lesions have also been treated successfully with surgical excision, as in our patient. Great care must be taken to avoid damage to orbital muscles, vessels, ocular nerves, and in particular the optic nerve, as the lesion in itself is benign and can be fully removed [16]. Prognosis after surgery is usually good. In our case, the ocular movements improved and double vision subsided as a result of orbital decompression and pressure release on the extraocular muscles. Additional steroids may accelerate recovery and are recommended in lower doses [2, 16].

In conclusion EH, although rare at this site, enters into the differential diagnosis of orbital masses. Histopathological examination is mandatory in order to exclude other tumours and tumour-like lesions. Despite being a benign process, its disrupting growth may cause severe symptoms, spontaneous regression is uncommon, and lesions often recur, so complete removal is the therapy of choice.
